# Radiation Pattern Prediction for Metasurfaces: A Neural Network-Based Approach

**DOI:** 10.3390/s21082765

**Published:** 2021-04-14

**Authors:** Hamidreza Taghvaee, Akshay Jain, Xavier Timoneda, Christos Liaskos, Sergi Abadal, Eduard Alarcón, Albert Cabellos-Aparicio

**Affiliations:** 1 NaNoNetworking Center in Catalonia (N3Cat), Universitat Politècnica de Catalunya, 08034 Barcelona, Spain; akshay1991jain@gmail.com (A.J.); xavitimonedacomas@gmail.com (X.T.); abadal@ac.upc.edu (S.A.); eduard.alarcon@upc.edu (E.A.); acabello@ac.upc.edu (A.C.-A.); 2 Foundation for Research and Technology Hellas, 71110 Heraklion, Greece; cliaskos@ics.forth.gr

**Keywords:** metasurface, machine learning, neural networks, beam steering, radiation pattern, 5G and beyond

## Abstract

As the current standardization for the 5G networks nears completion, work towards understanding the potential technologies for the 6G wireless networks is already underway. One of these potential technologies for the 6G networks is reconfigurable intelligent surfaces. They offer unprecedented degrees of freedom towards engineering the wireless channel, i.e., the ability to modify the characteristics of the channel whenever and however required. Nevertheless, such properties demand that the response of the associated metasurface is well understood under all possible operational conditions. While an understanding of the radiation pattern characteristics can be obtained through either analytical models or full-wave simulations, they suffer from inaccuracy and extremely high computational complexity, respectively. Hence, in this paper, we propose a neural network-based approach that enables a fast and accurate characterization of the metasurface response. We analyze multiple scenarios and demonstrate the capabilities and utility of the proposed methodology. Concretely, we show that this method can learn and predict the parameters governing the reflected wave radiation pattern with an accuracy of a full-wave simulation (98.8–99.8%) and the time and computational complexity of an analytical model. The aforementioned result and methodology will be of specific importance for the design, fault tolerance, and maintenance of the thousands of reconfigurable intelligent surfaces that will be deployed in the 6G network environment.

## 1. Introduction

Sixth-generation (6G) wireless networks will be even more heterogeneous and dense as compared to fifth-generation (5G) and other legacy networks. Thus, the 6G architecture will need to be adapted to serve the ever-evolving capacity and quality of service requirements [1,2]. To satisfy these ever-increasing demands, multiple enablers, such as visible light communication [3], light fidelity [4], Reconfigurable Intelligent Surfaces (RISs), TeraHertz (THz) communications, etc., have been proposed. Amongst these techniques, RISs have gained special attention. The reason being, through rapid tuning of the associated metasurfaces (MSFs), they transform the physical environment from being an adversary to being an ally in the communication process. Concretely, they enable the operators to engineer the channel propagation characteristics [5,6,7,8,9]. Such functionalities will be critical towards meeting the requirements being laid out for 6G networks [3,10,11].

Specifically, RISs, through their programmable characteristics, can perform the fine-grained manipulation of the radio signals being generated by the myriad transmitter devices/access points for their corresponding receivers. Such manipulations include absorption of certain components of the impinging radio signals, as well as fine-grained control of these signals in terms of direction, polarization, phase, and power in a frequency-selective manner [12,13,14,15]. Moreover, research efforts presenting experimental prototypes, such as [16,17,18], further provision insights into how the RISs can engineer the channel and demonstrate the feasibility of the RIS approach and its significant improvements for wireless communications in complex transmission environments. RISs also find their application in other use-cases such as the indoor geo-localization, which has been elaborated in [19,20,21]. Note that indoor geo-localization will be extremely critical for future networks as the use of mmWaves/THz band will necessitate detailed knowledge of the mobile device’s location for precise beamforming/beam-steering.

Further, the associated MSFs in RISs are electromagnetically thin-film and planar artificial structures that have resulted in the realization of Electromagnetic (EM) and optical components with engineered and even atypical functionalities [22,23,24,25]. In modern communication systems, MSFs have lots of applications such as phase correction [26], beam reconfigurability [27], and spatial filter [28]. MSFs can be made of all-dielectric structures such as printed layers of substrates [29] or all-metal structures [30].

It must be noted that the RIS consists of a device that controls the behavior of the EM waves, alongside devices that provide the tuning mechanism and the intelligence to control it. Furthermore, this device that controls the EM wave behavior can be realized using an MSF. Hence, the MSF is a component of the RIS. On a more granular level, an MSF is composed of subwavelength building blocks known as unit cells or meta-atoms. In this study, we also consider the case of tunable MSFs. From a general modeling perspective, unit cells consist of tunable resistors, *R*, and capacitances, *C*. This allows the unit cells to take multiple states and grants the MSFs their tunability characteristics. Notably, given a fixed EM functionality, the design of a static MSF is a complex task. Thus, the design and operation of a tunable MSF will be even more challenging. A significant development in this regard has been made through multiple research efforts, such as [9,31,32], in which possible architectures and characteristics of such programmable MSFs are discussed.

**Figure 1:**
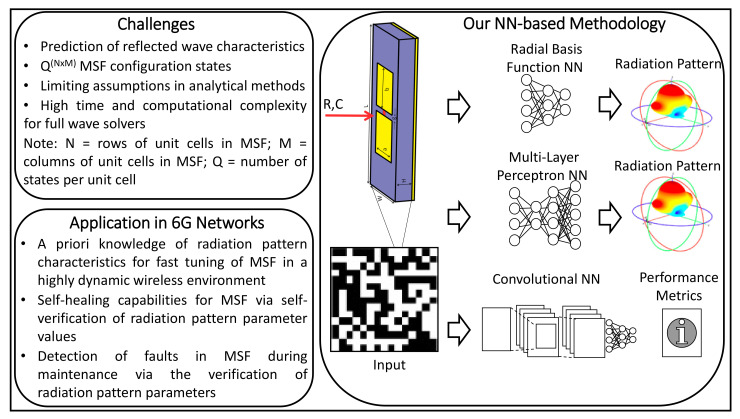
Neural Network-based approach for radiation pattern prediction: Challenges, Methodology and Application.

Besides, while tunability is an advantageous property of the programmable MSFs, an important challenge associated with them is obtaining the characteristics of a reflected wave given the parameters of the incident wave and the states of each composing unit cell. As illustrated in Figure 1, a fast yet accurate estimation of the radiation pattern will facilitate multiple applications for 6G networks, such as the design, reliable functioning, and maintenance of MSFs. Unit cells are multi-layer structures and in some cases posses embedded tuning elements for reconfiguration. Therefore, computing the characteristics of the reflected wave, given an MSF configuration, is a challenge. The reason being that they are obtained by either utilizing analytical methods with multiple limiting assumptions or by conducting computationally intensive simulations through full-wave EM solvers.

Knowing the EM characteristics of each unit cell facilitates the calculation of the corresponding EM field. In most cases, the unit cell and thus the MSF is reflective (the transmission coefficient is zero). Therefore, we just need to possess reflective features (reflection amplitude and phase) of the unit cell to estimate the far-field pattern. Analytical models exist for describing and predicting the reflected EM field in some well-defined cases, such as beam steering [33] and focusing [34] of planar impinging waves. Still, these models introduce simplifications which can result in limitations in realistic setups and, consequently, reduced precision of results compared to the direct solution from Maxwell’s equations [35]. Moreover, the iterative numerical full-wave simulations, which are widely adopted today and provide accurate predictions [36], are severely memory and time-consuming. Additionally, the design process largely relies on empirical reasoning or trial-and-error [37], which is inefficient and often ineffective.

On the other hand, machine learning (ML) techniques, and particularly Neural Networks (NNs), owing to their ability to learn complex relationships between input and output data, are capable of solving differential equations, thereby circumventing the need for numerical calculations [35,38,39]. This fact provides the intuition towards another direction: Since the MSF EM response is essentially the solution to Maxwell’s differential equations, it could be possible to design an ML construct that predicts the EM response much faster. In Table 1, we have compared calculation time of the EM response for different methods. It can be seen that NNs take around a minute to compute the EM response, whereas full-wave simulators such as CST Microwave Studio take almost one hour to compute the same field with the same resolution. Note that we utilize the MSF design and NNs studied in this work as well as the CST simulator to determine the order of computation time. It is important to state that, the numbers can vary depending on the dimension of MSF and configuration of NNs, but the order of magnitude will remain similar. Moreover, this has been corroborated by multiple studies such as [40,41,42]. For a more in-depth analysis into the computational complexity of NNs, analytical methods, and full-wave simulators, we refer the reader to Appendix B.

RIS empowered with artificial intelligence is capable of different functionalities such as programmable holography and focusing. While many works use RIS in optimized but random-looking configurations [16] and sometimes truly random configurations [20], we emphasize a relevant application for wireless communications (i.e., beam steering) to maintain the communication link for a moving target. Proposed NN models the far-field radiation pattern and/or metrics. However, the dimensionality is large, and thus, completely random patterns will lead to random scattering patterns. Training the NN for all of them ends up with overfitting and complicates the training process. Moreover, we are interested in directive beams; hence, it is reasonable to discard chaotic radiation patterns. Nevertheless, for the sake of generality, we do not discard all the random inputs. Instead, we use an entropy control to guarantee a representative portion of random-looking and non-random-looking configurations. This approach not only speeds up the training but also reduces the Mean Squared Error (MSE).

Overall, this work provisions a data-driven NN-based approach for determining an accurate estimation of the radiation pattern or performance metrics that enable the full characterization of the radiation pattern. As an example in Figure 1, the MSF is represented by the reconfiguration pattern, and unit cells are modeled with the ON-OFF states. We study the performance of our data-driven methodology using different NNs, i.e., Radial Basis Function NN (RBFNN), Multi-layer Perceptron NN (MLPNN), and the Convolutional NN (CNN). We describe the structure and functioning of these NNs in more detail in Section 4. We now elaborate on the salient contributions of this paper, as follows:

- We develop an NN-based radiation pattern predictor, which, through our analysis, is established to be nearly as accurate as a full-wave simulation but with the computational complexity of an analytical method.
- To the best of our knowledge, this is the first method wherein certain important features of the reflected beam radiation pattern for a given MSF, i.e., *Directivity, Principal-to-side-lobe ratio, Direction of maximum energy radiation* and *Half power beam width*, have been predicted and effectively utilized for the complete characterization of the reflected beam radiation pattern. Consequently, this also provisions the capacity of our methodology in 6G networks (Figure 1).
- We provide an analysis based on the accuracy of prediction of the aforesaid parameters, for the locally tunable MSF scenario. Through the incremental design methodology, we establish a concrete framework and benchmark towards the selection of a CNN-based predictor for the reflected beam radiation pattern. Specifically, we compare the performance of a CNN-based predictor with an MLP based predictor. The comparative study reveals that the CNN predictor provisions an accuracy similar to the MLP predictor. It is imperative to state here that a CNN incurs significantly lower computational complexity as compared to an MLPNN. To this end, we have also provided a short discussion on the computational complexity of the NN models, analytical method, and the CST full-wave simulator in Appendix B.

The remainder of this paper is organized as follows: In Section 2, we present the current state of the art. In Section 3, we describe the incremental design framework, including the multiple scenarios that we have analyzed. In Section 4, we elaborate upon the methodology that we have utilized for evaluating the multiple scenarios studied. In Section 5, we evaluate the NNs, and we conclude the paper in Section 6.

## 2. State of the Art

**Figure 2:**
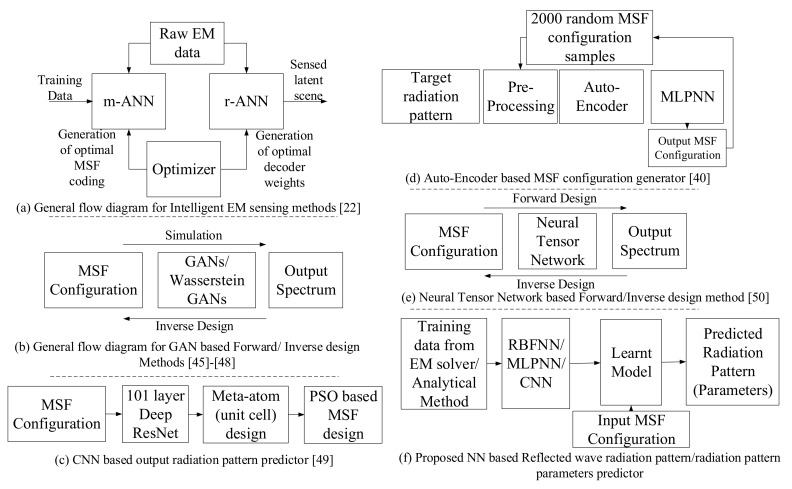
Schematic representation of state of the art approaches and the proposed method.

ML methods over the past decade have gained significant importance in multiple sectors such as aerospace, medicine, and telecommunications [43,44,45]. Further, since the laws of electromagnetism, fluid and aerodynamics are governed by well-known differential equation sets, the success of ML techniques in such domains is prospective [35,38,39]. Recently, several works in the research community proposed ML-based algorithms to design and validate EM response of MSFs [22,41,46,47,48,49,50,51]. Additionally, there are other data-driven approaches, such as the Particle Swarm Optimization (PSO) method for designing a wide range of MSFs. For example, PSO-based methods are used for designing artificial magnetic conductors, designing time-delay equalizer MSF for EM band-gap resonator antenna, and realization of a low profile bandpass frequency selective surface [52]. The aforesaid approaches are consolidated into a schematic diagram and are compared with our proposed method in Figure 2. Furthermore, we have organized the existing approaches into two distinct categories, i.e., *forward design approaches* and *inverse/MSF design approaches*. Whilst *forward design approaches* consist of methods wherein the MSF coding is used to predict the reflected wave radiation pattern, the *inverse/MSF design approaches* utilize the reflected wave radiation pattern as a feedback to optimize the MSF coding.

### 2.1. Forward Design Approaches

In Reference [50], an evolutionary algorithm that generates cell configurations and evaluates the fitness of each configuration by predicting the reflection phase with a trained CNN (a 101 layer deep residual network) for its given specific pattern has been proposed (Figure 2c). However, this CNN, which serves as a speedup of the optimization process of the evolutionary algorithm, is trained by previously encoding the output phases into a one-hot vector of length 360. Each element of the vector represents a discrete degree. Consequently, a problem that is purely based on regression is now converted into a classification problem. This results in a loss of resolution and thus crucial information with regards to the order and distance between degrees. Furthermore, in [50], the proposed CNN approach only provides good results for output radiation patterns with one, two, or three beams. Therefore, to use this approach as a reflected phase predictor, the user needs to know a priori how many lobes the resulting pattern will have, and given the fact that a method to a priori deduce the number of lobes has not been proposed in the aforementioned work, it thus limits the ability to generalize this approach to predict any reflected beam pattern.

Notably, other works, such as [53], have used ML tools for solving different EM problems as a replacement of conventional numerical simulations. In Reference [53], an encoder–decoder structure was employed for inferring the internal fields of arbitrary three-dimensional discretized nanostructures.

### 2.2. Inverse/MSF Design Approaches

In Reference [22], the authors propose a smart EM sensing mechanism, wherein the MSF coding pattern, as well as the information decoding parameters, are jointly optimized (deduced) to extract the latent scene (human gesture) information. To perform the same, the authors propose to utilize two deep NNs and an optimizer (Figure 2a). The deep NNs are termed as measurement ANN (m-ANN) and reconstruction ANN (r-ANN), wherein the m-ANN employs two CNNs. Further the m-ANN, in collaboration with the optimizer, determines the optimal MSF coding pattern. The r-ANN, on the other hand, employs three CNNs, and in coordination with the optimizer identifies the latent scene information from the received EM data. Next, in [46,47,48,49], Generative Adversarial Networks (GANs) have been used to solve the inverse problem, i.e., to determine the MSF unit cell structure, given the desired frequency response (Figure 2b). Additionally, in [46], a CNN is utilized as a simulator to verify the accuracy of the frequency response of transmittance from the generated structures during the training phase of the GANs generator component. Similarly in [47], a GAN-based simulator, faster than the conventional numerical simulation tools, has been proposed. This simulator is one of the components of a system that performs an inverse design to select an MSF pattern from a user-defined dataset to match the required input optical spectrum. Additionally, in [48], GANs have been employed to design the MSFs that can generate complex tensorial Radio Frequency (RF) responses. Further, and similar to previous methods, it also uses a CNN-based simulator to validate the RF response of the generated MSF configurations. Concretely, the CNN simulates and generates the scattering parameters for a given unit cell shape. However, the proposed simulator does not evaluate the complete radiation pattern of a locally or globally tunable MSF. Lastly, amongst the GAN-based methods, Ref. [49] works with a variant of the conventional GANs, i.e., Wasserstein GAN (W-GAN), to achieve its goal of identifying the most suitable MSF design.

Further, other research efforts such as [41] and [51] exploit other deep learning techniques to perform the task of MSF design. Concretely, in [41], an auto-encoder based approach has been adopted (Figure 2d). In this method, the auto-encoder enables capturing the most significant aspects of the input data, i.e., the desired reflected beam radiation spectrum. Subsequently, it facilitates the fully connected MLP network in determining the requisite MSF profile for the demanded radiation pattern. Moreover, in [51], a combination of the traditional NN, such as MLP, and Neural Tensor Network (NTN) based approach has been adopted for designing the MSF (Figure 2e). However, to do the same, an initial simulation framework consisting of two NNs, which employs the NTN, has been prepared. These NNs aim to predict the amplitude and phase of the reflected wave from the MSF. This is performed by using these two NNs to predict the real and imaginary part of the desired EM response, respectively. Following this accurate prediction, inverse design methodologies are then adopted to formulate MSFs conforming to a wide variety of design objectives, thus highlighting the versatility of the proposed approach.

Given the aforesaid studies, to the best of our knowledge, we claim that for estimation of the reflected wave radiation pattern our methodology provides a *simpler* solution. This can be highlighted from the fact that the study in [22] utilizes a set of two different deep ANNs, both comprising multiple CNN layers. Specifically, the m-ANN, which provisions the MSF coding for the optimal illumination of the field of interest, applies two deep CNNs. The CNN we use in the third scenario may end up generalizing to an NN with a similar architecture than m-ANN, but in this paper, we prove that with a simpler CNN architecture, we can obtain relevant features of the radiation pattern instead of the full radiation pattern. Moreover, in this study, MSF coding pattern is optimized for a specific application (shaping planar incident wavefront) while we conducted a general study (i.e., prediction of radiation pattern or performance metrics for any reconfiguration pattern). Next, the models defined in studies [46,47,48,49] work with GANs/W-GANs. These are again more complex DNN architectures as compared to the relatively simple CNN framework that we propose. Further, in [46,47,48,49], the objective is to study the inverse problem, i.e., to determine the MSF configuration from a set of input radiation patterns. Hence, such solutions do not apply to the objective of the problem that we aim to solve in this work. Moreover, the work done in [50] results in a loss of resolution and applies to only some well-known cases. Additionally, in [41,51], the proposed methodologies either suffer from scalability issues (MLP is not scalable for large MSF configurations) or develop multiple complex DNN architectures (such as two DNNs with the first layer being an NTN), respectively. Hence, from Figure 2f, it can be observed that proposed methodology, which we will detail next, is unique compared to the state-of-the-art approaches (Figure 2a–e) in terms of its structure and approach towards predicting the output radiation pattern/parameters. Table 2 lists the state-of-the-art papers comparing design approach, application, and year of the publication.

## 3. Incremental Design Framework

We now describe the framework for our radiation pattern predictor, wherein we consider two broad scenarios, i.e., homogeneous and heterogeneous MSF configurations, and incrementally demonstrate that it is possible to predict the features of the reflected wave from a given MSF configuration through data-driven learning approaches. Note that depending on the scenario, the MSF is represented by a matrix of unit cell states. Since the unit cells are not extremely small compared to the wavelength ($\lambda_{0}/3$) and phase reflection is the only parameter that is different for each unit cell, the coupling effect between the unit cells is negligible. The accuracy of this approximation is in excellent agreement with full-wave simulations [33,36,42] and has been used in different analyses [54,55,56].

Next, the homogeneous MSF configuration scenario is further expanded to two specific scenarios. These scenarios are established based on the underlying unit cell configurations of the MSF and are listed as follows:

- The non-tunable scenario consists of a non-tunable unit cell configuration across the MSF. Such a configuration is termed a non-tunable MSF.
- The globally tunable scenario consists of a matrix of unit cells across the MSF, wherein the unit cells have the same values for the tunable resistance *R* and capacitance *C*. Such a configuration is termed a globally tunable MSF.

**Figure 3:**
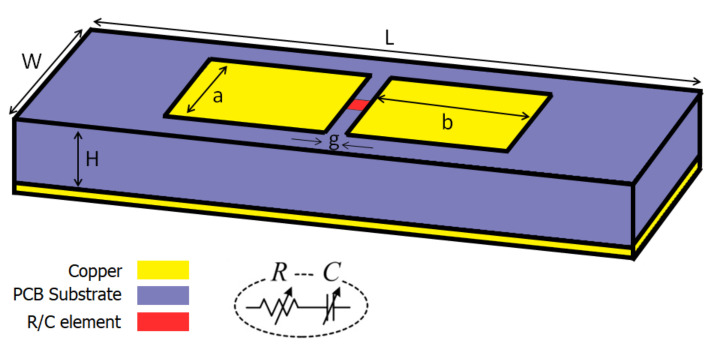
Schematic of the unit cell for the proposed MSF. The dimensions are L=30 mm, W=12 mm, H=3.18 mm, a=7.85 mm, b=7.50 mm and g=1 mm.

The unit cell design is a modified version of the previous work [57] which can be a tunable perfect absorber for different incidence angles and have tunable anomalous reflection toward different directions at 5 GHz. Figure 3 presents the schematic of the unit cell in which two metallic patches are connected via tunable *R* and *C* elements. The PCB thickness is 3.18 mm, the relative permittivity is ϵ=2.2, and loss tangent is δ=0.0009. A plane wave impinges on the unit cell. Then, s by comparing the reflected wave’s amplitude and phase, reflection coefficients of the unit cell are computed. This is computed with full-wave simulation.

**Figure 4:**
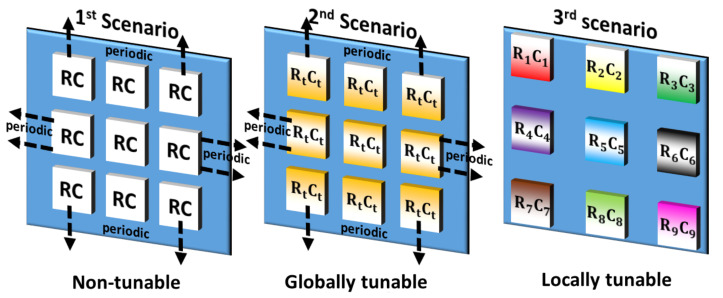
Diagram of the three scenarios utilized in the Incremental Design framework. Non-tunable and Globally tunable scenarios correspond to the broader homogeneous MSF configuration category, while the locally tunable scenario corresponds to the heterogeneous MSF configuration category.

Subsequently, the heterogeneous MSF configuration scenario, or the locally tunable MSF, refers to the scenario where the unit cells can have different values of *R* and *C* associated with them. An illustration of these three scenarios that we have analyzed in this work is presented in Figure 4. It is worth stating here that in our framework, we analyze the efficacy of NN-based approaches by starting from a simple scenario, i.e., the non-tunable (static) MSF, to a more generic and complex scenario, i.e., the locally tunable (Programmable) MSF. Hence, we now describe these scenarios and the associated methodologies for radiation pattern prediction in detail through Section 3 and Section 4, respectively.

### 3.1. Homogeneous MSF Configuration

In this scenario, we elaborate upon the two scenarios, i.e., the non-tunable MSF and the globally tunable MSF, in the text that follows.

#### 3.1.1. Non-Tunable Scenario (Non-Tunable, Single Unit Cell/Full Radiation Pattern Estimation)

Interaction coefficients for the general analysis of MSFs can be very complicated [58]. Note that such MSFs can be used for focusing [59] and polarization selection [60]. Next, based on the Floquet theory, we can approximate large enough (>>$\lambda_{0}/2$) periodic structures with an infinite array of the same unit cell configuration (Figure 4, Case 1). This is because the fields propagate only with phase delays together with multiplication by periodic coefficients. This translation is considered automatically with the usage of periodic boundary conditions in the simulations, thus reducing the complexity. This is a very extended practice in this kind of study [61]. Thereby, we just consider one unit cell and apply periodic boundary conditions to model the whole MSF. Plus, in the prediction process, it is presumed that the azimuth and elevation angles of the incident EM wave are given.

#### 3.1.2. Globally Tunable Scenario (Tunable Single Unit Cell/Full Radiation Pattern Estimation)

In this scenario, we introduce a data-driven model to predict the complete reflected wave radiation pattern for a globally tunable MSF. One of the reasons for studying the globally tunable MSF configurations is the role that they will play in applications such as perfect absorber [62], frequency-tunable absorber [63], amplitude modulator [64] and polarization control [65]. Same as before, in this scenario the MSF consists of an infinite array of unit cells, wherein the same tuned unit cell configuration is repeated *ad infinitum* (Figure 4, Case 2).

### 3.2. Heterogeneous MSF Configuration

#### 3.2.1. Locally Tunable Scenario (Tunable Full Surface/Radiation Pattern Attribute Estimation)

Here, we elaborate upon the locally tunable scenario, which expands our incremental framework to a locally tunable MSF (Figure 4, Case 3). Such MSFs enable applications such as object tracking [19,20,21] and sensing [22,24]. Hence, they will be of significant importance in 6G networks.

In this scenario, the inputs for our NN-based framework are two-dimensional matrices, with each value representing the 8 possible states of the unit cell at the corresponding position in the MSF. Additionally, the corresponding MSF is a 12×12 matrix of unit cells. The framework thus attempts to predict, for normal incident angles, the measures of the reflected beam radiation pattern for an MSF with a set of given unit cell state configurations. Note that the number of possible configurations for the MSF under study, is $8^{144}$. Due to the large dimensionality, we investigate a data-driven model to predict four measures of interest that characterize the reflected wave radiation pattern instead of the radiation pattern itself. These measures of interest are the *Directivity*, *Principal-to-side-lobe ratio*, *Angle of maximum radiation* and *Half power beam width*.

## 4. Methodology

Training an NN with environmental parameters like the location and geometry of the obstacles requires specific information. Simulation of this complex medium is a challenge and modeling it with analytical methods is not accurate. Therefore, in this framework, we assumed the RIS in free space whereas the environment does not affect the RIS response (i.e., objects are not in the reactive near-field of the RIS). Nevertheless, this does not mean the scenario under study is unrealistic because the input of the NN is the reconfiguration profile of the MSF, and the output is the radiation pattern. Therefore, by learning this relationship between the reconfiguration profile and the MSF response, we can manipulate the radiation pattern to optimize the communication channel. Moreover, free space RIS was the subject of several recent works such as free space optical systems [66], smart radio environments [67,68,69], vehicular communication systems [70,71], and a line of sight wireless communications [5]. Regardless of the actual characteristics of the source (e.g., horn antenna), far-field impinging waves upon the MSF can be considered to be locally planar [69]. Therefore, we assumed a plane wave with a normal incident that illuminates the MSF area uniformly.

### 4.1. Homogeneous MSF Configuration

#### 4.1.1. Non-Tunable Scenario

For the simulations in the non-tunable scenario, we sweep the azimuth and elevation angles from 0 to 89 degrees with respect to normal incidence direction alongside a resolution of 1 degree. Given that the transmittance is 0, we do not need to evaluate negative elevation angles. Moreover, due to the assumed unit cell symmetries, we do not need to explore all the azimuth angles.

The NN model that we explore for our data-driven framework is RBFNN. It is a three-layer fully connected NN, wherein the inputs from the input layer are fed to a hidden layer via weighted links. At the hidden layer, a euclidean distance between the input and the link weight vector, also known as neuron’s center, is computed [72]. The activation function of the neurons is the Gaussian function, and these are also known as the basis functions [72]. Consequently, the output of the neurons in the hidden layer is determined by the output of the Gaussian function operated over the distance between the input and neuron’s center. The final output is obtained by combining the weighted outputs of the neurons in the hidden layer [72]. Such a paradigm is a priori very interesting for our approach since it models spatial variables. This is in contrast to the MLPNN, in which the basis functions are based on the dot product. Concretely, this enables the RBFNN to learn the non-linear relationship between the incident and reflection angles of the EM wave more effectively than an MLPNN, which is inherently based on a linear transformation.

Note that for the accuracy of evaluation of the RBFNN, we set the MSE goal to be $10^{-11}$ and the spread constant to 1. The spread constant here refers to the spread (or variance) of the Gaussian radial basis functions. This hyper-parameter consequently plays a crucial role in determining how the input layer is mapped onto these basis functions [73]. Further, 8100 samples were collected using an EM simulator, which in this work is the CST Microwave Studio, of which 85% were spend for training and validation and the rest, i.e., 15%, for evaluating the model generalization (which is usually referred to as the testing process). For non-deep learning scenarios, the aforementioned set of hyper-parameters lies within the range of values that are chosen usually [50].

#### 4.1.2. Globally Tunable Scenario

In the globally tunable scenario, we vary the values of the parameters that characterize the physical structure of the unit cell, i.e., resistance *R* and capacitance *C*. However, as described earlier, the entire MSF consists of the same unit cell configuration throughout, i.e., all tuned unit cells will have the same value for *R* and *C*. For brevity, we studied only normal incident wave direction. However, if required, our model can be extended to any incident wave direction (incident angle). Further, we sweep the values of *R* from 1 Ω to 100 Ω with a resolution of 1 Ω, and that of *C* from 0.1 pF to 1 pF with a resolution of 0.01 pF. The framework we use in this scenario for our data-driven approach is the MLPNN.

Moreover, unlike scenario 1, wherein the spatial characteristics of the incidence and reflected angles of the impinging wave were to be learned, in scenario 2, the input features R and C lack any spatial characteristics. Thus, we do not evaluate RBFNN for this case. Furthermore, and owing to its relatively poor performance in scenario 1, we do not explore CNN for scenario 2.

For the MLPNN, we utilize a single hidden layer of 20 neurons. The training algorithm used was scaled conjugate gradient [74] without any regularization. The non-requirement of any regularization in our model was due to the fact that it has a very small amount of parameters. Similar to the non-tunable scenario, we obtained the samples from an EM simulator and delimited 85% of them for training and the rest for testing purposes. We collected 9191 samples, which is slightly more than the number of samples collected for the non-tunable scenario.

### 4.2. Heterogeneous MSF Configuration (Locally Tunable Scenario)

**Figure 5:**
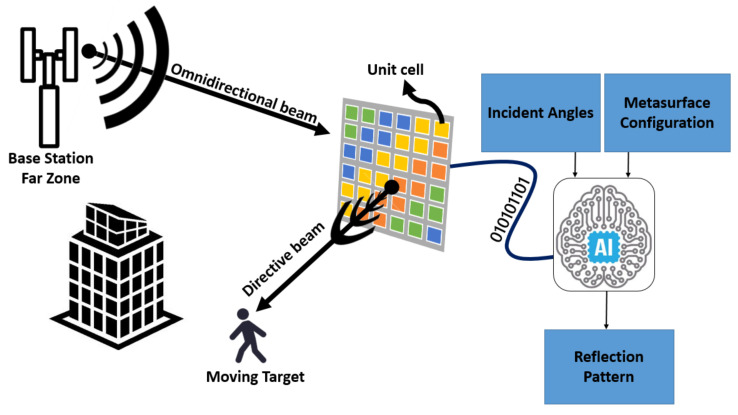
Graphical exhibition of the system model: Base station at the far zone radiates with an omnidirectional pattern. Planar impinging wave on the MSF reflected toward the target with a precise configuration of the MSF imposed by the well-trained NN.

As mentioned in Section 3.2.1, for locally tunable MSF (Figure 5), we have to deal with a large input dimensionality ($8^{144}$). To train an NN and test it, we need numerous input data ($10^{6}$). Since unit cells are tuned differently, it is not possible to adopt periodic boundary condition. This increases the computation time. Given our computational limitations, collecting enough samples through an EM simulator would take an extremely long time. Therefore, in addition of hundreds of simulated samples, we collected thousands of samples through an analytical method. With this technique, we trained an NN as fast as analytical solutions (c.f. Appendix B) and as accurate as EM solvers (c.f. Section 5). Therefore, in this paper, and for this scenario, we demonstrate that

- Our ML approach predicts the measures of the reflected beam pattern accurately.
- Provided that there is enough computational power, we can extrapolate the same model and methodology to the scenario where we have more samples from an EM solver.

Analytical Model—Fourier transform can be used to estimate the MSF radiation properties for simple cases in which 1-bit coding governs two states ∈[0,1] representing full reflection (PEC) and no reflection [75]. We can model the current distribution on the unit cells with complex shapes [76] and even use circuit modeling to imitate the feed [77]. However, we do not need such complexity for unit cell patches. In our case, the omnidirectional radiation pattern from the far-field becomes planar after traveling for a couple of wavelengths distance [69]. Hence, MSF is illuminated by an incident plane wave. Knowing the incident angles, i.e., the elevation angle $\theta_{i}$ and the azimuth angle $\phi_{i}$, we can engineer the direction of reflection by an appropriate linear phase gradient [57,78,79]. Assuming that the MSF imposes the phase profile Φ(x,y), we assign the virtual wave vector $k_{\Phi }=\nabla \Phi =\partial_{x}\Phi \;\hat{x}+\partial_{y}\Phi \;\hat{y}$ ($\partial_{x}$ and $\partial_{y}$ denote partial derivatives). The momentum conservation law can then be expressed as [36]

(1) $$\begin{array}{c} k\sin\theta_{i}\cos\varphi_{i}+\partial_{x}\Phi =k\sin\theta_{r}\cos\varphi_{r}\\ k\sin\theta_{i}\sin\varphi_{i}+\partial_{y}\Phi =k\sin\theta_{r}\sin\varphi_{r} \end{array}$$

where $\partial_{x}\Phi$ and $\partial_{y}\Phi$ describe the imposed phase gradients in the *x* and *y* directions, respectively, and the subscripts *i* and *r* denote incident and reflected (scattered) waves, respectively. Assuming air as the transmission medium, the required phase shift then reads

(2) $$\Phi_{ij}=\frac{2\pi D_{u}\left(i\cos\varphi_{r}\sin\theta_{r}+j\sin\varphi_{r}\sin\theta_{r}\right)}{\lambda_{0}}$$

Next, given that the size of the unit cells in an MSF is small, surface current on them can be accurately modeled as a collection of sources [33]. Then, we can compute the reflected wave radiation pattern by applying the Huygens principle and the principle of superposition of waves, wherein the far-field is the sum of the contributions of all unit cells. For linearly polarized incidence, the scattered field can be expressed as [80].

(3) $$\begin{array}{c} E\left(\theta ,\varphi \right)=\sum_{i=1}^{M}\sum_{j=1}^{N}A_{ij}e^{j\alpha_{ij}}f_{ij}\left(\theta_{ij},\varphi_{ij}\right)\\ \Gamma_{ij}e^{j\Phi_{ij}}f_{ij}\left(\theta ,\varphi \right)e^{jk_{0}\zeta_{ij}\left(\theta ,\varphi \right)} \end{array}$$

where *M* and *N* are the number of unit cells in a row or a column, $k_{0}$ is the wave number, φ and θ are the azimuth and elevation angles, $A_{ij}$ and $\alpha_{ij}$ are the amplitude and phase of the wave incident on the ij-th unit cell, $\Gamma_{ij}$ and $\Phi_{ij}$ are amplitude and phase reflection coefficient for the ij-th unit cell, and $f_{ij}$ denotes the scattering pattern of the ij-th unit cell, which, according to reciprocity, is identical for scattering toward the (θ,φ) direction and the interception of incoming waves from the $\left(\theta_{ij},\varphi_{ij}\right)$ direction. Here, we assume $f_{mn}\left(\theta ,\varphi \right)=\cos\left(\theta \right)$ which describes real-world dipolar scatterers. Finally, $\zeta_{ij}\left(\theta ,\phi \right)$ is the relative phase shift of the unit cells with respect to the radiation pattern coordinates (ϕ,θ) [42,80],

(4) $$\zeta_{ij}\left(\theta ,\phi \right)=D_{u}\sin\theta \left[\left(i-\frac{1}{2}\right)\cos\phi +\left(j-\frac{1}{2}\right)\sin\phi \right]$$

Training and Testing dataset generation—Next, for ML, normally random selection is used to generate samples for training. However, random inputs of the gradient for unit cells will always end up in a random scattering pattern. These patterns, in addition to being non-learnable, will not be of significance for design purposes. Thus, the samples collected for training are not entirely random combinations within the whole space, wherein the total number of combinations, as mentioned earlier, is $Q^{\left(N\times M\right)}=8^{144}$.

Additionally, in our approach, a sample generation space is defined to control the entropy of the input data [75]. Specifically, low entropy regimes will only be useful to train specific options, i.e., those that require ordered codings (focusing, beam steering), but they will not suffice scenarios that need near-to-random codings (Radar Cross Section (RCS) reduction). Hence, a wide entropy range, such as the one as we have described above, will be essential to train the NN and enables it to generalize effectively. Using a simple algorithm to generate random samples for MSF configuration, there is a huge chance to get high entropy data. In most cases, the products of the algorithm are unrelated configurations. Due to the huge number of possibilities, we cannot iterate the data generation frequent enough to make sure the low entropy data is produced as well. Here, we introduced a method to overcome this issue. In this procedure, a configuration is acquired with Equation (2) for a random pair of reflection angles. Thereafter, an entropy factor is introduced into the matrix of configuration with an adjustable size that allows us to control the entropy of the final configuration ranging from 0 to 100%. With this method, generated data includes configurations with uncorrelated data with high entropy as well as meaningful configurations with low entropy. Therefore, we obtained a vast range of entropy with a reasonable number of iterations (e.g., $10^{6}$) which is precisely what we need to prevent overfitting.

Subsequently, a criterion on the radiation pattern (e.g., Directivity) can be applied to discriminate interpretative configurations. This criterion translates the qualification of the NN on a specific configuration. Therefore, we have a system that automatically checks if the new configurations used for predicting its measures can be used (with a reasonable granted accuracy) in the NN for prediction.

Adopting the aforementioned procedure, the number of samples that were collected for training and testing the model of the locally tunable scenario was $10^{5}$. Amongst these samples and similar to the first two scenarios, 85% of them were used for training and validation whereas the remaining 15% were stored in a completely separate set for the testing phase of the model. Further from the training and validation set, 80% of the samples were used for training, while the remaining 20% were used for validation. The values of the pixels in the input images were normalized by performing a max-min escalation, without modifying their variance. This is not the case for our input variables, as the variance of each pixel is part of the relevant information the model uses for prediction.

However, it is important to state that standardizing the features is important when we compare measurements that have different units, as variables that are measured at different scales will not contribute equally and could end up creating a bias, and since this is the case for the target variables, the target samples for both training and testing sets were standardized by subtracting the mean of each of the measures and dividing them by their respective variances.

**Figure 6:**
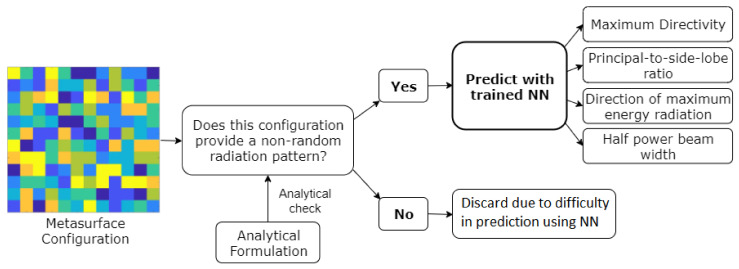
Diagram of the steps performed inside the system once the model is trained for the locally tunable scenario.

Prediction System Operation—Following this, once our model is trained for a given upcoming configuration, it performs an analytical check of the given configuration and determines if it is totally random or not. If after the analytical check it is determined that the MSF configuration is not totally random, the trained model is used to predict the measures of interest. Instead, if it is determined otherwise during the analytical check, meaning that the configuration outputs a random radiation pattern, it is discarded as the model cannot provide suitable results for this configuration. Concretely, since NN training is time-consuming, inputs that are not suitable for the application under study are removed. Moreover, and as we have already stated, we employ the entropy control methodology for ensuring diversity in the inputs for NN training. However, during the prediction stage, configurations that are not suitable for the specified application are eliminated via the analytical check. Figure 6 illustrates the aforesaid steps performed in our system for predicting the measures of interest from an upcoming MSF configuration, once the model is trained.

NN Models—With this background, we now delve deeper into the setup of the two NN models that we use for our evaluations within the locally tunable scenario. Note that for this scenario, unlike the non-tunable scenario of the homogeneous MSF configuration (Section 4.1.1) where the spatial characteristics of the incidence and reflected angles were to be learned, we do not use the RBFNN since we are not considering any spatial characteristics in our input space.

#### 4.2.1. Multi-Layer Perceptron Neural Network

As part of our methodology, illustrated in Figure 6, we adopt NNs for predicting the measures of interest of the reflected beam radiation pattern. Note that we consider measures of interest here as against the full radiation pattern, given the prohibitively large nature of the search space in highly scattering environments for predicting the complete reflected wave radiation pattern. Next, in this section, we consider MLP as our candidate NN. For the MLP case, the input images of 12×12 pixels which represent the unit cell configurations are flattened into vectors of 144 variables before being introduced into the NN.

**Figure 7:**
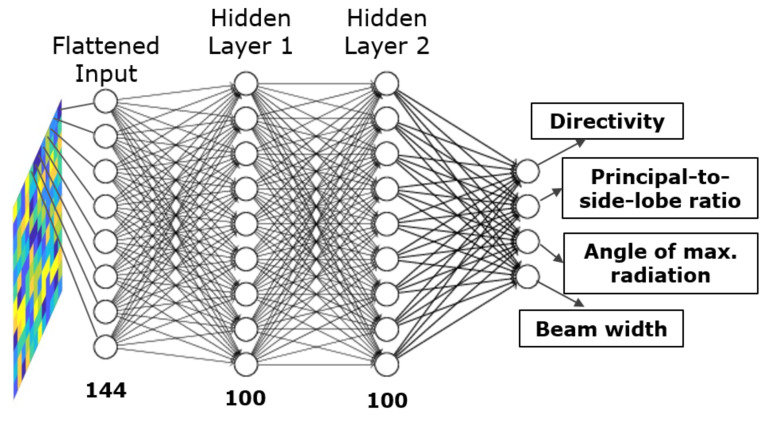
Structure of the Multi-Layer Perceptron Neural Network in the locally tunable scenario.

Figure 7 shows the structure of the MLPNN approach for the locally tunable scenario. The number of hidden layers and the neurons per layer was set to 2 and 100, respectively. A conclusion, with regards to the aforesaid parameter values, was reached after extensive user-driven exploration since sweeping across all the possible combinations was not computationally feasible. The rest of the parameters for the MLPNN are listed in Table 3.

As we can observe from Table 3, the training algorithm selected is the scaled conjugate gradient which accelerates the convergence rate for first-order algorithms, like the steepest descent, while avoiding the high computational cost of second-order methods, such as Newton’s method. Since the default learning rate parameter worked reasonably well for the NN training, i.e., it provided a reasonable convergence time and performance, we did not deem it necessary to tune it further. Whilst this could be a point of optimization, we leave it for a future work as our objective in this paper is to demonstrate the efficacy of the method.

Next, regularization is a way to limit the complexity of a model and hence reduce the chances of overfitting by penalizing the most complex solutions in the cost function. Thus, we employ an L2 regularization in our methodology. Specifically, in the model, this is enforced via the λ hyper-parameter. For a more detailed discussion on the regularization aspect, regularization method selection, and the associated hyper-parameter value selection in our model, we refer the reader to Appendix A.

#### 4.2.2. Convolutional Neural Network

Another NN that we explore for our methodology is the CNN. For the CNN case, the input images of 12×12 pixels and additionally a channel, which represents the unit cell configurations, are directly introduced to the NN.

**Figure 8:**
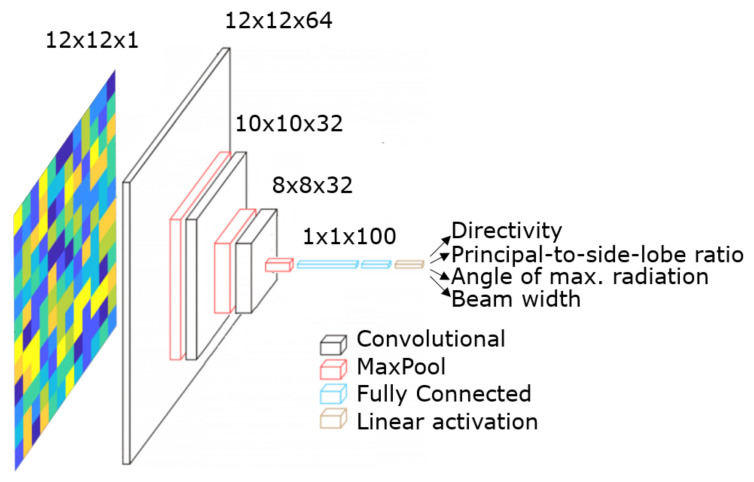
Structure of the Convolutional Neural Network in the locally tunable scenario.

Figure 8 illustrates the structure of the CNN-based approach for the locally tunable scenario. It is composed of three convolutional layers that consist of 64, 32, and 32 filters, respectively. Further, a max-pooling process is performed after each of them. For all the convolutional layers, the filter size is 3×3 pixels, and the stride is 1. As we do not use zero paddings, the dimensionality of the intermediate images which represent the activations is reduced at each layer. They are followed by a fully-connected layer with 100 neurons and an output layer with a linear activation function. Similar to the MLPNN case, the architectural parameters of the CNN are a result of extensive user-driven exploration, since sweeping around all the possible combinations was not computationally feasible. We enlist the most significant CNN architecture-related parameters in Table 4.

As we can observe, the training algorithm selected is the stochastic gradient descent (Table 4). As it is a first-order optimizer, the steps of the optimization process are linearly done concerning the direction of the maximum gradient. Thus, the length of the steps needs to be defined by the learning rate hyper-parameter. The learning rate, decay, momentum, and the number of both convolutional and fully connected layers, specified in Table 4, are set following a user-driven exploration. It is important to state here that we do not use zero paddings as it would introduce noise to the data, by essentially forcing a boundary that would be non-existent on a continuous MSF plane.

Additionally, the third convolutional layer and the fully connected layer are regularized through a dropout process. This process consists of randomly ignoring a given number of layer outputs during the training process. Therefore, the layer with the dropout process is treated as a layer with a lower number of nodes and connectivity to the previous layer. In effect, each update to a layer during training is performed with a different “view” of the configured layer. Dropout factor controls the number of nodes and is ignored randomly. For the third convolutional layer and the fully connected layer, the dropout factors are 0.2 and 0.25, respectively. These values were selected following the same procedure explained for selecting the λ regularization parameter in the MLP.

## 5. Evaluation

Given the framework discussed in Section 3 and Section 4, we now present the evaluation for each of the scenarios discussed within this framework and highlight the relevant outcomes and insights. The results obtained from our NN-based prediction system have been compared to the ground truth results obtained via the CST simulator.

### 5.1. Homogeneous MSF Configuration

#### 5.1.1. Non-Tunable Scenario

**Figure 9:**
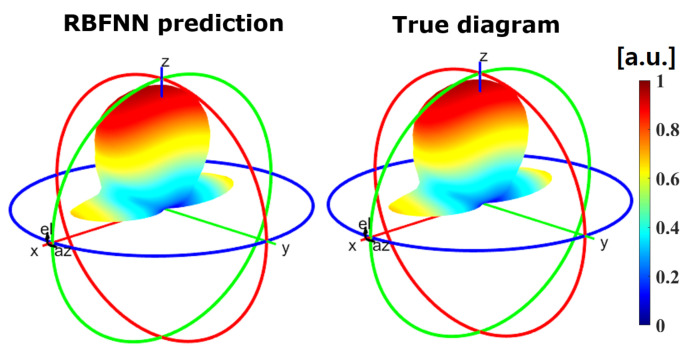
Comparison between the predicted normalized radiation pattern by the RBFNN of the non-tunable scenario (**left**) and the true diagram (**right**) for azimuth an elevation values of 89.5 and 88.7 degrees with respect to the normal direction, respectively.

For the non-tunable, single unit cell/full radiation pattern case, the trained RBFNN was able to predict the radiation pattern for any given angle of incidence with an $R^{2}$ test of 0.9994. Therefore, this assists us in validating our hypothesis that ML models can accurately predict the reflected wave radiation pattern from a single unit cell for every angle of the incident wave. Figure 9 illustrates a visual comparison between the predicted radiation pattern by the trained RBFNN and the true diagram obtained through EM simulation, for the azimuth and elevation angles that were not present in the training set. Therefore, our prediction system can accurately learn and generalize for untrained/unseen angles within the training dataset.

Further, when a CNN was applied for this case, the observed MSE was $10^{-7}$, which is significantly worse as compared to the accuracy obtained via the RBFNN approach (the MSE goal to measure the RBFNN accuracy was set to $10^{-11}$). Hence, for the sake of brevity, for scenario 1 we only highlight the results from the evaluations carried out using the RBFNN approach.

#### 5.1.2. Globally Tunable Scenario

**Figure 10:**
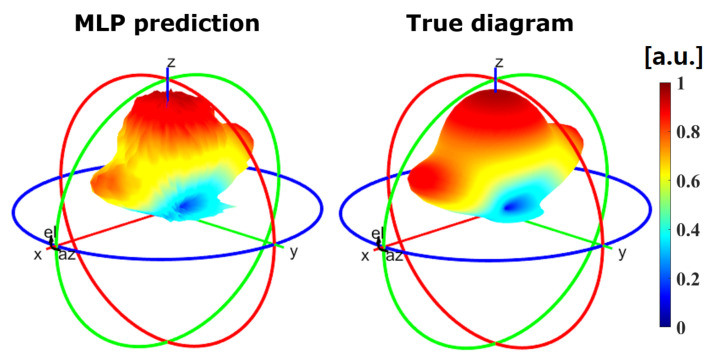
Comparison between the predicted normalized radiation pattern by the MLP of the Globally tunable scenario (**left**) and the true diagram (**right**) for R and C values of 2.5Ω and 0.25 pF, respectively.

For the tunable, single unit cell/full radiation pattern case, the trained MLP was able to predict the radiation pattern for any given R and C value with an $R^{2}$ test of 0.9849. Therefore, our hypothesis that ML models can accurately predict the radiation pattern of the reflected wave in a single unit cell for each R and C combination has also been validated. Figure 10 shows the visual comparison between the predicted radiation pattern by the trained MLPNN and the true diagram obtained through EM simulation, for R and C values that were not present in the training set. This reinforces the fact that our predictor can learn and generalize to scenarios with untrained/unseen values of R and C within the training dataset.

Further, here, we do not present a discussion of results for this scenario with the RBFNN and CNN setups. Specifically, given that RBFNN is not suitable for the globally tunable scenario, and the CNN performs poorly for the non-tunable scenario, we do not detail a discussion on the performance of these setups here.

### 5.2. Heterogeneous MSF Configuration (Locally Tunable Scenario)

The radiation pattern *attribute* prediction problem for the locally tunable scenario is essentially a regression problem. Hence, the cost/error function to minimize during the training process is the MSE. However, this error function does not provide a very good interpretability of the performance. Alternatively, we define a tolerance (or a set of tolerances) specific for each measure of interest. Subsequently, we evaluate the percentage of the predictions that fall within this tolerance limit. This is termed as the accuracy measure in this paper. Thus, in the following Section 5.2.1, Section 5.2.2, Section 5.2.3 and Section 5.2.4, we discuss the performance of the MLPNN and CNN over the different measures of interest that we aim to predict using our methodology ((Figure 4). The results associated with the ensuing discussions are reported in Table 5.

#### 5.2.1. Directivity

For *Directivity*, we observed that the MLPNN provided near-perfect prediction, subject to certain tolerance limits. Concretely, from Table 5, it can be seen that 95% of the test samples have been accurately predicted when the tolerance is set to 0.25 dB. Moreover, when the tolerance is relaxed further, i.e., to 0.5 dB, we observe an improved accuracy of 99.99%. However, when the tolerance limit is reduced to 0.1 dB, we notice that the accuracy of the MLPNN degrades drastically to 56.3%.

On the other hand, when the CNN is used as the predictor, the accuracy of prediction with a 0.25 dB tolerance limit is set on 90.6% (Table 5). Further, when the tolerance is increased to 0.5 dB, the accuracy of prediction is improved to 99.8%. Additionally, when the tolerance limit is reduced to 0.1 dB, similar to the MLPNN case, the accuracy of prediction for the CNN is deteriorated significantly to 48.8%. These aforementioned accuracy measures are lower than those offered by the MLPNN. This is because, an MLP based method, due to the fully connected architecture, can learn almost any feature space accurately. On the other hand, a CNN tries to extract the most significant features through its convolution-based processing, and hence, it is a lossy method.

However, a point of contention with the MLPNN is that the fully connected architecture is not scalable for bigger MSF configurations. This will progressively become detrimental to the system performance, as the cost of computation will increase exponentially. In contrast, a CNN utilizes significantly less computational and memory resources and will scale better, whilst providing an accuracy measure that is close to that offered by the MLPNN.

#### 5.2.2. Principle-to-Side Lobe Ratio

For the *Principle-to-side lobe ratio*, we obtain similar observations from Table 5, as we did for the *Directivity* parameter. Specifically, for the MLPNN, when we vary the tolerance from 0.5 dB to 0.25 dB and finally to 0.1 dB, the corresponding accuracy measures are registered at 99.9%, 98.3%, and 86.1%, respectively. On the other hand, for the same tolerance value ensemble, the CNN method produces accuracy measures of 99.4%, 94.3%, and 80.1%, respectively.

Therefore, we can see that the MLPNN performs slightly better than the CNN. However, as mentioned earlier, this comes at a significant computational cost, thus hampering its scalability. Besides, it is understood that the correlation between the neighboring unit cells is far less as compared to those that are found in images in general [50]. Consequently, this corroborates the findings from Table 5, with regards to the CNN performing slightly worse as compared to the MLPNN. Concretely, an MLPNN can learn the interactions between the distinctly related neighboring unit cells much more effectively due to the fully connected architecture. However, a CNN treats the MSF like an image, thus considering the neighboring unit cells to be correlated. However, in reality, this is seldom the case.

It is imperative to state here that, the aforesaid non-relational nature of nearby unit cells is also responsible for the visibly subdued performance of the CNN, as compared to the MLPNN, for other measures of interest.

#### 5.2.3. Angle of Maximum Radiation

The results for the angle of maximum radiation in Table 5 are obtained by averaging the accuracy of prediction of the elevation and azimuth angles, to provide a single view over this feature. Subsequently, we observe that the MLPNN performs slightly better than the CNN, the reasons for which have been expressed in Section 5.2.2.

To elaborate, for this measure, we consider tolerance values of 5º, 2º, and 1º. Next, from Table 5 we observe that the MLPNN has an accuracy of 99.8%, 72.7%, and 40.6% for the corresponding tolerance values, respectively. Further, the CNN approach has an accuracy of 98.9%, 60.7%, and 31.9%, given the same tolerance value ensemble, respectively. As can be seen, the accuracy drops as we reduce the tolerance limit, which is in line with our observations from the other measures of interest. Additionally, it can be deduced that irrespective of the NN utilized for the prediction step, the accuracy for the lower tolerance values is significantly less as compared to the other measures of interest.

#### 5.2.4. Half Power Beam Width

For this measure, we consider the tolerance values of 1º, 0.5º, and 0.25º. From Table 5, we observe that the MLPNN has the corresponding accuracy measures of 99.5%, 97.3%, and 79.2%, respectively. Further, the CNN has accuracy measures of 98.8%, 92.6%, and 61.8%, respectively. The trend for the accuracy values is similar to that observed for the other measures of interest (Section 5.2.1, Section 5.2.2 and Section 5.2.3).

**Figure 11:**
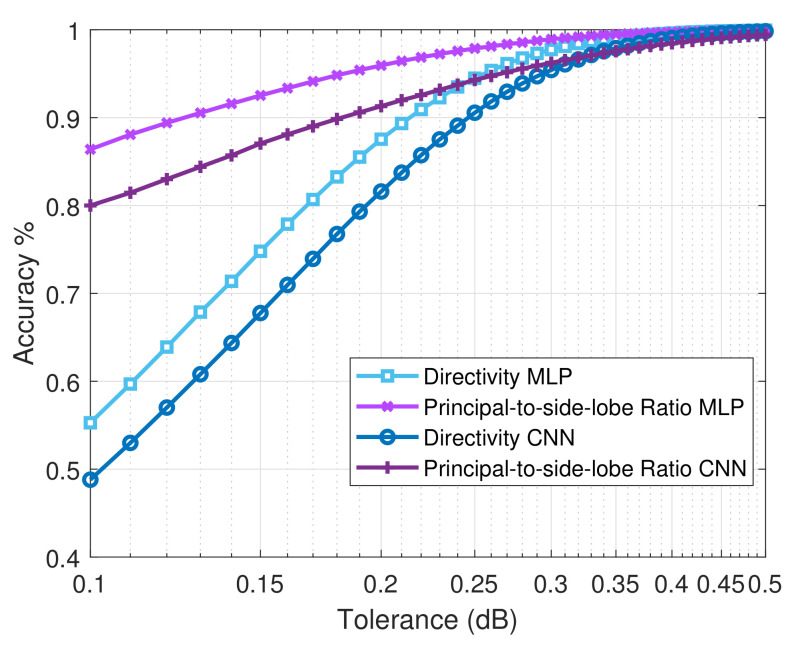
Accuracy vs tolerance in dB for both MLPNN and CNN. The curves shown correspond to Directivity and Principal-to-side-lobe Ratio.

**Figure 12:**
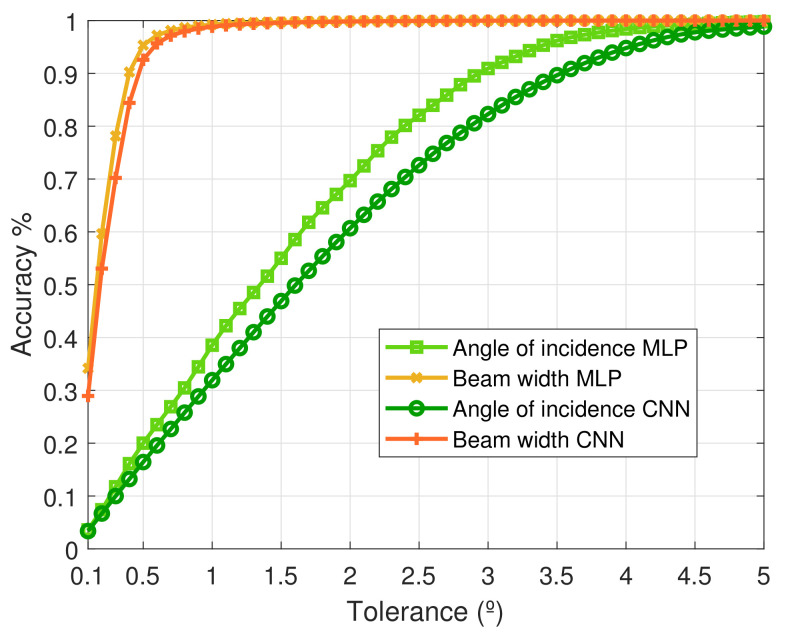
Accuracy vs tolerance in degrees for both MLPNN and CNN. The curves shown correspond to the Angle of maximum radiation and Beam width.

Hence, from the discussions so far, we can deduce that the proposed methodology can accurately predict the reflected beam radiation pattern or the measures that can fully characterize the same. To further reinforce this idea, we present Figure 11 and Figure 12. Concretely, Figure 11 shows in detail the evolution of the accuracy of the predictions for the *Directivity* and *Principal-to-side-lobe ratio* as the tolerance in dB grows, for both MLP and CNN cases. We observe that the trend for the accuracy is exactly what we have deduced through our discussions in Section 5.2.1, Section 5.2.2, Section 5.2.3 and Section 5.2.4. Further, we see that as the tolerance approaches 0.5 dB, the accuracy of the CNN predictor approaches that of the MLP.

Lastly, Figure 12 illustrates the evolution of the accuracy of the predictions for the *Angle of incidence* and *Half Power Beam Width* as the tolerance in degrees grows, for both MLP and CNN cases. Again, here we observe that the accuracy percentage improves as the tolerance is increased. However, we also notice that the Beam width prediction approaches near 100% accuracy at very low tolerance values, whilst the *angle of radiation* measure necessitates higher tolerance limits for the predictors to achieve better accuracy.

## 6. Conclusions

In this paper, we have presented a data-driven methodology, wherein we developed an NN-based approach for characterizing the reflected beam radiation pattern from an MSF. One of the most important advantages of such an approach is that, while its accuracy is close to the full-wave simulation, the time complexity to achieve the same is significantly smaller. Additionally, it can also serve as a methodology that enables self-healing characteristics and facilitates maintenance aspects of MSFs in the 6G wireless network environment.

As part of this methodology, we have provisioned an incremental design framework. Through this framework, we analyzed three specific scenarios, wherein we estimated radiation pattern of non-tunable MSFs and globally tunable MSFs. Further, through our analysis, we have demonstrated the efficacy of the NN-based approaches. Concretely, it was observed that the NN-based approaches could predict the radiation pattern with very high accuracy in a significantly reduced time frame as compared to the full-wave simulator counterparts.

Moreover, through the locally tunable scenario, we demonstrated that our CNN-based prediction framework performs as well as the fully connected MLPNN framework. However, it does so with a significantly reduced computational complexity as compared to the fully connected MLPNN framework. This will especially be critical when the framework is scaled up to even larger MSF.

Further, through the last scenario, we have provisioned the first study, wherein, instead of estimating the entire radiation pattern, we have predicted the most important parameters that govern any radiation pattern, i.e., Directivity, Principle-to-side lobe ratio, Angle of maximum radiation, and Beam width. This process will not only ensure the required reliability in estimation, but it will also allow for a faster convergence time for such estimations.

Since the objective of this paper was to understand the feasibility of the discussed approaches, only a simulation-based analysis was performed. Hence, as part of future work, a more realistic framework based on true data (measurement) should be carried out.

## Figures and Tables

**Table 1:** Estimation of computation time for radiation pattern calculation with different methods> For general overview refer to Appendix B.

Methods	Computation Time
Analytical Methods	∼1 s
Full-Wave Simulators	∼1 h
Neural Networks	∼1 min

**Table 2:** Summarizing the state of the art data-driven approaches.

Design Approach	Application	Year	Reference
Optimizer	Time-delay equalizer	2017	[52]
Two Deep NN and optimizer	Smart sensing	2020	[22]
GAN and CNN	Frequency response prediction	2018	[46]
GAN	Inverse design	2019	[47]
GAN and CNN	MSF design	2019	[48]
GAN	MSF design	2021	[49]
CNN	Reflection phase prediction	2019	[50]
Auto-encoder	MSF design	2019	[41]
MLP and NTN	MSF design	2019	[51]
Encoder-decoder	Field prediction	2020	[53]

**Table 3:** Multi-Layer Perceptron Neural Network parameters.

Parameter Name	Value
Regularization type	L2
λ	0.8
Training algorithm	scaled conjugate gradient
Number of hidden layers	2
Neurons of 1st hidden layer	100
Neurons of 2nd hidden layer	100

**Table 4:** Convolutional Neural Network architecture parameters.

Parameter Name	Value
Regularization type	Dropout
Dropout factor 3rd conv. layer	0.2
Dropout factor FC layer	0.25
Training algorithm	Stochastic Gradient Descent
Learning rate	0.001
ineMomentum	0.9
Decay	$10^{-4}$
Num. of conv. layers	3
Num. of FC layers	1

**Table 5:** Accuracy Measure: MLPNN vs CNN.

Parameter	MLPNN		CNN	
	Tolerance	Accuracy	Tolerance	Accuracy
Directivity	0.5 dB	0.999	0.5 dB	0.998
	0.25 dB	0.950	0.25 dB	0.906
	0.1 dB	0.563	0.1dB	0.488
Principle-to-side lobe ratio	0.5 dB	0.999	0.5 dB	0.994
	0.25 dB	0.983	0.25 dB	0.943
	0.1 dB	0.861	0.1 dB	0.801
Angle of maximum radiation	5${}^{\circ }$	0.998	5${}^{\circ }$	0.989
	2${}^{\circ }$	0.727	2${}^{\circ }$	0.607
	1${}^{\circ }$	0.406	1${}^{\circ }$	0.319
Half Power Beam Width	1${}^{\circ }$	0.995	1${}^{\circ }$	0.988
	0.5${}^{\circ }$	0.973	0.5${}^{\circ }$	0.926
	0.25${}^{\circ }$	0.792	0.25${}^{\circ }$	0.618

## Data Availability

Not applicable.

## Appendix A

In the MLP case, the typical regularization methods are L1 and L2. These methods add a new term in the cost function that sums all the non-zero weights. The significance of this term is governed by the regularization parameter λ, and while the main advantage of L1 regularization is that it forces sparsity into the models by forcing most of the weights to zero, for our case, we do not require feature selection since the number of input pixels is quite limited. Consequently, L2 regularization is selected, and the new loss function is defined as

$$L\left(X,y\right)=MSE\left(X,y\right)+\lambda \sum_{i=1}^{N}w_{i}^{2}$$

where *X* represents the input images, *y* the target metrics, λ the regularization parameter, N the total number of neurons of the MLP, and *w* each weight of all the layers.

Furthermore, for selecting the optimal L2 regularization parameter λ, we performed 10-fold cross-validation. For each parameter value, we split the dataset into 10 groups, and for each group, we train a model with the remaining 9 groups and validate with the selected group. Then, the validation errors of each combination are averaged. Finally, the parameter value that provides the lowest cross-validation MSE is selected for the final model.

## Appendix B

Considering the size of the MSF as an N×M matrix of unit cells and the MLPNN configuration in Section 3, the complexity of executing the MLPNN is O(NMη), where η is the number of neurons in the first layer of the MLPNN. Next, for the CNN architecture (Section 4) the complexity is O(NMpq), where *p* and *q* are the width and height of the filter utilized in the convolutional layer. It is imperative to state here that, in general, pq<<η. In this work, p=q=3 and η=100, thus provisioning two orders of magnitude difference between the product pq and η. On the other hand, for the analytical model specified in Equation (1) (Section 4.2), the computational complexity is O(NM). Moreover, practical implementation defines that the values of *p* and *q* are usually chosen as 3 or 5 [81]. Hence, from the aforesaid complexity analysis and the NN models used in our work, it is clear that the CNN approach achieves complexity close to the analytical approach whereas the complexity of the MLPNN is at least two orders of a magnitude greater than that of the analytical and CNN model. Furthermore, for the MSF size considered in our work the CST full-wave simulator needs nearly 2 million mesh blocks to be computed. Concretely, this translates to more than $O(NM\cdot 10^{4})$ computations. Thus, the CST full-wave simulator requires nearly 4 and 6 orders of magnitude more computations as compared to the NN models utilized in this work.
